# Probiotic Mixture of *Lactobacillus plantarum* Strains Improves Lipid Metabolism and Gut Microbiota Structure in High Fat Diet-Fed Mice

**DOI:** 10.3389/fmicb.2020.00512

**Published:** 2020-03-26

**Authors:** Huizhen Li, Fei Liu, Jingjing Lu, Jialu Shi, Jiaqi Guan, Fenfen Yan, Bailiang Li, Guicheng Huo

**Affiliations:** ^1^Key Laboratory of Dairy Science, Ministry of Education, Northeast Agricultural University, Harbin, China; ^2^College of Food, Northeast Agricultural University, Harbin, China

**Keywords:** *Lactobacillus plantarum*, obesity, lipid accumulation, lipid metabolisim, gut microbiota, short chain fatty acids

## Abstract

The global prevalence of obesity is rising year by year, which has become a public health problem worldwide. In recent years, animal studies and clinical studies have shown that some lactic acid bacteria possess an anti-obesity effect. In our previous study, mixed lactobacilli (*Lactobacillus plantarum* KLDS1.0344 and *Lactobacillus plantarum* KLDS1.0386) exhibited anti-obesity effects *in vivo* by significantly reducing body weight gain, Lee’s index and body fat rate; however, its underlying mechanisms of action remain unclear. Therefore, the present study aims to explore the possible mechanisms for the inhibitory effect of mixed lactobacilli on obesity. C57BL/6J mice were randomly divided into three groups including control group (Control), high fat diet group (HFD) and mixed lactobacilli group (MX), and fed daily for eight consecutive weeks. The results showed that mixed lactobacilli supplementation significantly improved blood lipid levels and liver function, and alleviated liver oxidative stress. Moreover, the mixed lactobacilli supplementation significantly inhibited lipid accumulation in the liver and regulated lipid metabolism in epididymal fat pads. Notably, the mixed lactobacilli treatment modulated the gut microbiota, resulting in a significant increase in acetic acid and butyric acid. Additionally, Spearman’s correlation analysis found that several specific genera were significantly correlated with obesity-related indicators. These results indicated that the mixed lactobacilli supplementation could manipulate the gut microbiota and its metabolites (acetic acid and butyric acid), resulting in reduced liver lipid accumulation and improved lipid metabolism of adipose tissue, which inhibited obesity.

## Graphical Abstract

The mixed lactobacilli (*Lactobacillus plantarum* KLDS1.0344 and *Lactobacillus plantarum* KLDS1.0386) prevented high fat diet-induced obesity via regulating gut microbiota and lipid metabolism of the adipose tissue and inhibiting liver lipid accumulation in mice.

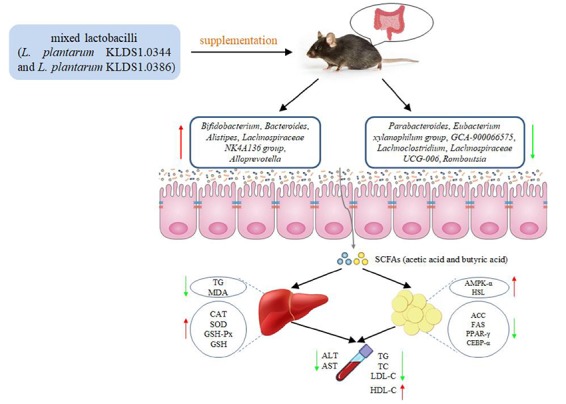

## Introduction

Obesity has become a worldwide epidemic disease and has a serious impact on the healthy development of the human body, which is a major hidden danger to public health (Risk and Factor Collaboration, 2016; Lu J. et al., 2016; Solas et al., 2017). The researchers collected data from 195 countries and found that the prevalence of obesity worldwide has increased dramatically since 1980 (Reuter and Mrowka, 2019). In 2015, approximately 603 million adults and 107 million children suffered from obesity, with an overall prevalence of 12% for adults and 5% for children (GBD 2015 Obesity Collaborators, 2017). Obesity is defined by the World Health Organization as an excessive accumulation of fat that may be harmful to health and is diagnosed when body mass index (BMI) ≥30 kg/m^2^ (Prospective Studies and Collaboration, 2009). In general, the intake of a large amount of high-sugar and high-fat food, sedentary work style and less physical exercise cause the energy intake to be larger than the energy consumption of the body, which will lead to the accumulation of excessive triglyceride in liver, kidney week and adipose tissue, thus causing obesity (Hurt et al., 2010; Heymsfield and Wadden, 2017; Bluher, 2019). Obesity can increase the risk of various diseases such as cardiovascular disease (Oikonomou and Antoniades, 2019), type 2 diabetes (Dietz, 2017), osteoarthritis (Schott et al., 2018), Alzheimer’s disease (Solas et al., 2017), anxiety (Ogrodnik et al., 2019), depression (Tyrrell et al., 2018), and certain cancers [for example breast cancer (Picon-Ruiz et al., 2017; Hao et al., 2018), colorectal cancer (Wunderlich et al., 2018), pancreatic cancer (Zaytouni et al., 2017), stomach cancer (Murphy et al., 2018) and liver cancer (Shin et al., 2013)].

At present, the medications approved by the US Food and Drug Administration (FDA) for long-term weight management mainly reduce energy or food intake by causing fat malabsorption, promoting satiety, delaying gastric emptying, decreasing appetite, or acting on the central nervous system pathway, but they all have certain side effects such as nausea, diarrhea, vomiting, dry mouth, constipation, dizziness and fecal incontinence (Davidson et al., 1999; Smith et al., 2010; Gadde et al., 2011; Apovian et al., 2013; Xavier et al., 2015). For some patients with obesity to a certain extent, gastric banding, Roux-en-Y gastric bypass and vertical-sleeve gastrectomy, three types of surgery for obesity, are common treatments (Neylan et al., 2016; Schauer et al., 2016). Although more effective than drug intervention, these procedures are more risky (American College of Cardiology/American Heart Association Task Force on Practice Guidelines Obesity Expert Panel, 2013, 2014) and may cause some complications (Almalki et al., 2017). Therefore, safer and healthful non-drug therapies have been proposed, including the use of probiotics. *Lactobacillus rhamnosus* Lb102 and *Bifidobacterium animalis ssp. lactis* Bf141 isolated by Le Barz et al. from fermented milk products and human feces, respectively, were used to interfere with mice fed with high-fat diet and it was found that they could effectively alleviate the onset of obesity and reduce the content of liver fat in mice (Le Barz et al., 2019).

*Lactobacillus plantarum* KLDS1.0344 and *L. plantarum* KLDS1.0386 were isolated from traditional fermented dairy products in Inner Mongolia, China, and preserved in our laboratory. In addition, our previous studies have demonstrated that *L. plantarum* KLDS1.0344 and *L. plantarum* KLDS1.0386 have strong acid and bile salt resistance, high cell adhesion activities and lipid metabolism regulation properties (Tang et al., 2016; Jin et al., 2017; Lu et al., 2018; Yan et al., 2019; Yue et al., 2019). Hence, we investigated whether a mixture of *L. plantarum* KLDS1.0344 and *L. plantarum* KLDS1.0386 could prevent obesity. The results showed that the combined treatment of *L. plantarum* KLDS1.0344 and *L. plantarum* KLDS1.0386 could significantly improve some obesity-related indicators in high fat diet-fed mice, including body weight gain, Lee’ s index, and body fat rate, etc. which established its effect in inhibiting obesity (Lu J. et al., 2019). However, the mechanism of the combined treatment of *L. plantarum* KLDS1.0344 and *L. plantarum* KLDS1.0386 to inhibit obesity was still unclear. Therefore, the aim of this study was to further explore whether the combined intervention of *L. plantarum* KLDS1.0344 and *L. plantarum* KLDS1.0386 prevented obesity by reducing liver lipid accumulation and regulating lipid metabolism and gut microbiota.

## Materials and Methods

### Materials

Triglyceride (TG), total cholesterol (TC), low density lipoprotein cholesterol (LDL-C), high density lipoprotein cholesterol (HDL-C), antioxidant enzymes, glutathione (GSH), malondialdehyde (MDA), alanine aminotransferase (ALT) and aspartate aminotransferase (AST) assay kits were obtained from Nanjing Jiancheng Bioengineering Institute (Nanjing, China). PrimeScript^TM^ RT reagent Kit with gDNA Eraser was obtained from Takara Biomedical Technology (Beijing) Co. Ltd. (Beijing, China).

### Preparation of Bacterial Strains

*Lactobacillus plantarum* KLDS1.0344 and *L. plantarum* KLDS1.0386 were inoculated in De Man Rogosa Sharpe (MRS) broth, respectively, according to 2% inoculation amount, cultured at 37°C, subcultured every 24 h, cultured in the third generation to 18 h, then centrifuged at 2500 *g* at 4°C for 10 min to harvest bacterial cell, and washed with a sterile phosphate buffered saline (PBS) for three times. The washed *L. plantarum* KLDS1.0344 and *L. plantarum* KLDS1.0386 were separately resuspended with sterile PBS to reach a concentration of 5 × 10^8^ CFU/mL, and then the two were mixed at a ratio of 1: 1. Bacteria were freshly prepared daily during the 8-week experiment.

### Experimental Animals

Male Specific Pathogen-Free (SPF) C57BL/6J mice, aged 21–28 days, were provided by Beijing Vital River Laboratory Animal Technology Co. Ltd. (Beijing, China) (Approval No. SCXK (JING) 2012-0001). These mice were housed in plastic cages under environmentally controlled conditions (temperature, 20–22°C; relative humidity, 50 ± 10%; and lighting, 12 h light/12 h dark cycle) and given free access to standard diet and water to acclimate to the environment for a week before the start of the experiment. The animal experiment protocol was approved by the Institutional Animal Care and Use Committee of the Northeast Agricultural University under the approved protocol number Specific pathogen free rodent management (SRM)-06.

### Experimental Design

After one week of acclimatization, the mice were randomly assigned to the following three groups: control group (Control), high fat diet group (HFD) and mixed lactobacilli group (MX), with 8 mice in each group. Mice in the control group were fed D12450B control diet, while the others were fed D12492 high fat diet. The feed was manufactured and supplied by Beijing Keao Xieli Feed Co. Ltd. (Beijing, China), and its formula is shown in Supplementary Table S1. From 9: 00 to 11: 00 am every day, the control group and the high fat diet group mice were gavaged with 0.2 mL of sterile PBS solution, and for the mixed lactobacilli group, the mice was administered with 0.2 mL of the mixed lactobacilli suspension (10^8^ CFU). During the whole experiment, the padding and water were changed twice a week, and the high-fat diet was changed every day to prevent the oxidation of fat to produce odor which affected the mice to eat. The entire experiment lasted for 8 weeks. Eight weeks later, all mice were removed from diet for 12 h and then were anesthetized and sacrificed. The serum samples were obtained via centrifugation at 1500 *g* for 10 min at 4°C, and stored at −80°C for further analysis. Livers and epididymal fat pads were stored at −80°C. The cecal contents of the mice were collected into sterile tubes and stored at −80°C for later use.

### Determination of TC, TG, LDL-C and HDL-C in Serum

The concentration of TC, TG, LDL-C and HDL-C in serum of each group of mice were measured with the commercial assay kits (Nanjing Jiancheng Bioengineering Institute, Nanjing, China) according to the manufacturer’s protocols. The final data of TC, TG, LDL-C and HDL-C are reported as mmol/L.

### Determination of ALT and AST in Serum

The activity of ALT and AST in serum of each group of mice were measured with the commercial assay kits (Nanjing Jiancheng Bioengineering Institute, Nanjing, China) according to the manufacturer’s protocols. The levels of ALT and AST are reported as U/L.

### Determination of TG, Antioxidant Enzymes, GSH and MDA in the Liver

Liver homogenates were centrifuged at 1150 *g* for 10 min at 4°C and the protein concentration was measured by bicinchoninic acid (BCA) method using a commercial kit (Nanjing Jiancheng Bioengineering Institute, Nanjing, China). Then, the levels of TG, catalase (CAT), superoxide dismutase (SOD), glutathione peroxidase (GSH-Px), GSH and MDA in the mouse livers were measured using commercially available kits (Nanjing Jiancheng Bioengineering Institute, Nanjing, China) following the manufacturer’s instructions.

### Expression of Related Genes in Epididymal Fat Pads

To determine the expression of key genes for lipid metabolism in epididymal fat pads, mRNA levels of adenosine 5′-monophosphate-activated protein kinase-α (AMPK-α), hormone-sensitive lipase (HSL), peroxisome proliferators-activated receptor-γ (PPAR-γ), CAATT/enhancer binding proteins α (C/EBPα), fatty acid synthetase (FAS) and acetyl CoA carboxylase (ACC) were assessed by quantitative real-time polymerase chain reaction (qPCR). Total RNA of the tissues was extracted with TRIzol reagent (Invitrogen, Carlsbad, CA, United States) according to the manufacturer’s instructions. The mRNA was reverse transcribed into cDNA using the PrimeScript^TM^ RT reagent Kit with gDNA Eraser (Takara, Otsu, Japan). Quantitative real-time PCR was performed using reverse-transcribed cDNA as a template, and ABI7500 fluorescence quantitative PCR instrument (Applied Biosystems, Woolston, Warrington, United Kingdom) and SYBR Green PCR Master Mix (Applied Biosystems, Woolston, Warrington, United Kingdom) were used according to the manufacturer’s protocols. Specific forward and reverse primer sequences for quantitative real-time PCR are listed in Supplementary Table S2. All reactions were performed in triplicate. Relative quantification of gene expression were analyzed with the 2^–ΔΔ^ ^Ct^ method. The target gene levels were calculated relative to β-actin and the data were shown as fold changes.

### Analysis of Gut Microbiota

Total bacterial DNA of the cecal contents in each group (*n* = 3) was extracted using the E.Z.N.A.^®^ Stool DNA Kit (Omega Bio-Tek, Norcross, GA, United States) according to the manufacturer’s instructions. The extracted DNA was checked by agarose gel electrophoresis and quantified using a NanoDrop ND-2000 spectrophotometer (Thermo Fisher Scientific, Wilmington, DE, United States). The V3-V4 hypervariable region of the bacterial 16S rDNA was amplified by PCR using forward primer 338F (5′-ACTCCTACGGGAGGCAGCAG-3′) and reverse primer 806R (5′-GGACTACHVGGGTWTCTAAT-3′). The products of PCR were purified with AxyPrep DNA Gel Extraction Kit (Axygen Bioscience, Union City, CA, United States), quantified using Qubit 2.0 Fluorometer (Life Technologies, Carlsbad, CA, United States), and pooled in equimolar ratios. PCR amplicons sequencing was performed on Illumina Miseq platform (Illumina Inc. San Diego, CA, United States) following the standard protocols. The resulting raw reads were merged with FLASH software (V1.2.11) (Magoč and Salzberg, 2011) and quality-filtered using QIIME software (V1.7.0) (Caporaso et al., 2010). High-quality clean tags were obtained by using the UCHIME algorithm to identify and remove the chimeric sequences (Edgar et al., 2011). The tags with nucleotide 97% sequence identity were clustered into the same operational taxonomic units (OTUs) using Uparse software (V7.0.1001) (Edgar, 2013). These OTUs were subjected to analysis using the Greengenes database by PyNAST software (Version 1.2) and were annotated to taxonomic information (Yilmaz et al., 2013). The species abundance of microorganisms in the three groups at phylum and genus levels were compared. Linear Discriminant Analysis Effect Size (LEfSe) was used to identify potential microbial biomarkers associated with different treatments with an effect size threshold of 2 (Segata et al., 2011).

### Determination of Short Chain Fatty Acids (SCFAs) in the Intestine

In order to determine the level of SCFAs in the intestine, 50 mg the cecal contents were mixed in 0.3 mL pure water, treated with a ball mill at 45 Hz for 4 min, ultrasonically treated in ice water bath for 5 min, and centrifuged at 5000 *g* for 20 min at 4°C. The supernatant (0.2 mL) and 0.3 mL pure water were mixed evenly, treated with a ball mill at 45 Hz for 4 min, ultrasonically treated in ice water bath for 5 min, and centrifuged at 5000 *g* for 20 min at 4°C. After that, 0.3 mL the supernatant was uniformly mixed with the original 0.2 mL the supernatant, and 0.1 mL 50% H_2_SO_4_ and 0.4 mL the internal standard solution (2-Methylvaleric acid dissolved in diethyl ether to 50 μg/mL) was added. Next, the sample was centrifuged at 12000 *g* for 15 min at 4°C and allowed to stand at −20°C for 30 min. The supernatant was transferred to a sample vial and detected by gas chromatography-mass spectrometry (GC-MS). GC-MS detection was performed using an Agilent 7890 gas chromatography mass spectrometer equipped with an Agilent HP-FFAP capillary column (30 m × 250 μm × 0.25 μm, J&W Scientific, Folsom, CA, United States). Specific chromatographic conditions were used with reference to the method previously described (Zheng et al., 2013). Acetic acid, butyric acid, propionic acid, valeric acid (Sigma, St. Louis, MO, United States) were used as the standards. The concentration of each SCFA was determined according to a standard curve obtained from seven different concentrations of standards.

### Statistical Analysis

All experiments were performed with at least three replicates and all experimental data were displayed as mean ± standard deviation (SD). Analysis of the data was carried out using SPSS 20.0 software (SPSS Inc. Chicago, IL, United States). Statistical differences among groups were determined using one-way analysis of variance (ANOVA), followed by Duncan’s multiple range test. The Spearman’s rank correlation coefficients between the relative abundance of mixed lactobacilli-manipulated gut microbiome and obesity-related indicators were determined using R software 3.4.1 for correlational statistical analysis. *P*-values < 0.05 were considered to be statistically significant.

## Results

### Effect of Mixed Lactobacilli on Blood Lipids

**FIGURE 1:**
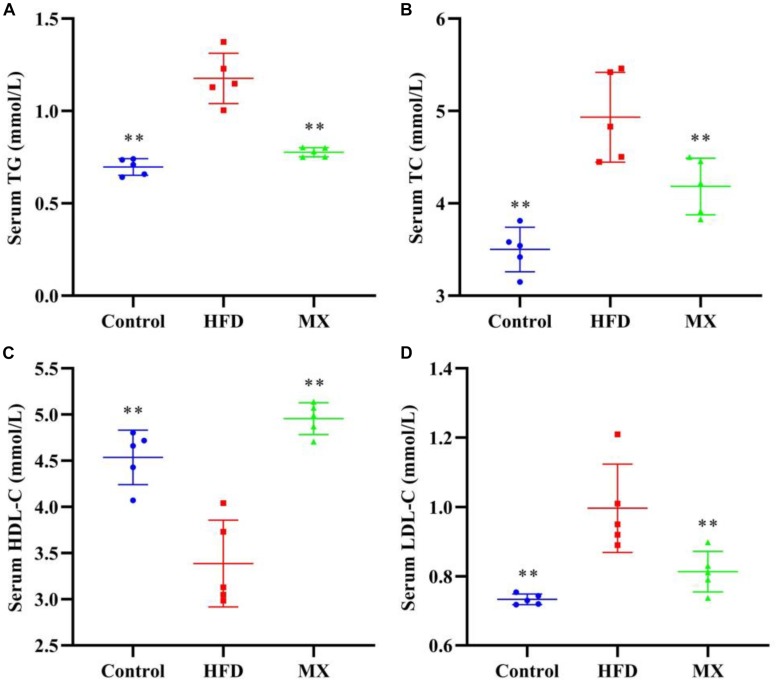
Effects of mixed lactobacilli on blood lipids. Control, control group; HFD, high fat diet group; and MX, mixed lactobacilli group. **(A)** Serum triglyceride (TG) level; **(B)** Serum total cholesterol (TC) level; **(C)** Serum high density lipoprotein cholesterol (HDL-C) level; and **(D)** Serum low density lipoprotein cholesterol (LDL-C) level. All data are represented as mean ± SD. **p < 0.01: significant difference compared with mice in the HFD group.

The blood lipid levels of the three groups of mice are shown in Figure 1. Compared with the control group, mice in the HFD group showed a dramatical increase in the serum levels of TG, TC, and LDL-C and significant decrease in the serum level of HDL-C (*p* < 0.01), indicating that abnormal blood lipid metabolism was occurred in the HFD group. Conversely, oral administration of mixed lactobacilli markedly inhibited these changes of lipid parameters in the serum (*p* < 0.01).

### Effect of Mixed Lactobacilli on Liver Function

**FIGURE 2:**
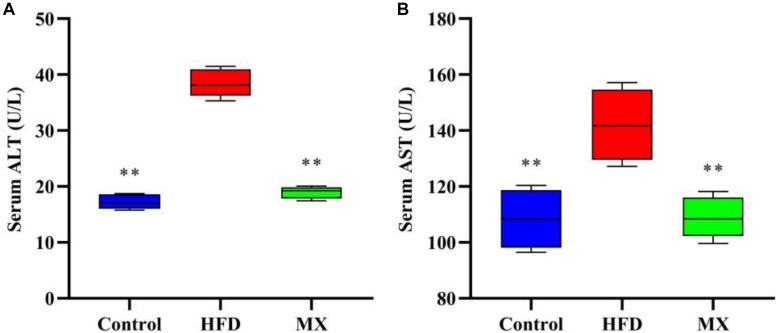
Effects of mixed lactobacilli on liver function. Control, control group; HFD, high fat diet group; and MX, mixed lactobacilli group. **(A)** Serum alanine aminotransferase (ALT) level; and **(B)** Serum aspartate aminotransferase (AST) level. All data are represented as mean ± SD. All data are represented as mean ± SD. **p < 0.01: significant difference compared with mice in the HFD group.

Serum ALT and AST levels, which are commonly used as indicators for evaluating liver function, were measured. As shown in Figures 2A,B, in a comparison between the control and HFD groups, the HFD group showed significantly higher levels of ALT and AST in serum than that of the control group (*p* < 0.01), indicating liver damage in these mice. Notably, treatment with mixed lactobacilli significantly reduced serum ALT and AST levels induced by high fat diet (*p* < 0.01).

### Effect of Mixed Lactobacilli on Lipid Accumulation in the Liver

**FIGURE 3:**
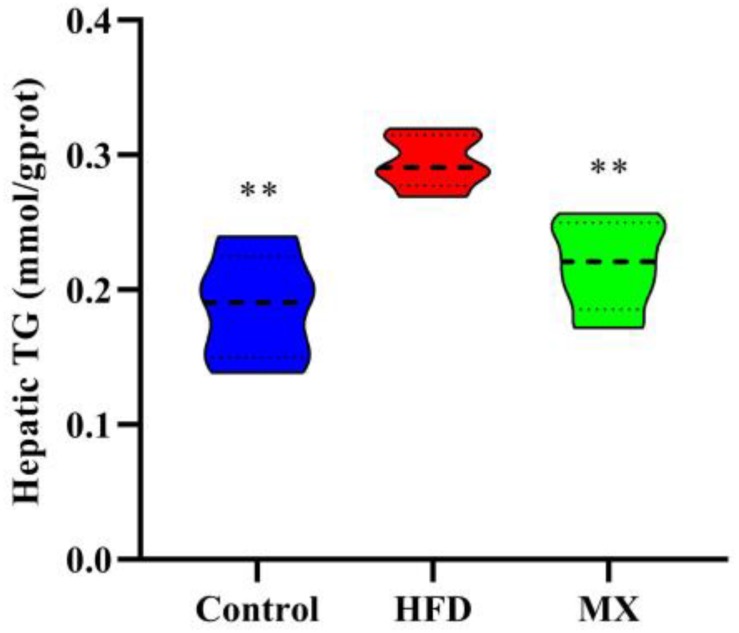
Effect of mixed lactobacilli on hepatic triglyceride (TG). Control, control group; HFD, high fat diet group; and MX, mixed lactobacilli group. All data are represented as mean ± SD. **p < 0.01: significant difference compared with mice in the HFD group.

To determine lipid accumulation in the liver of mice, we examined the levels of TG in the liver of the three groups of mice. As shown in Figure 3, the liver TG concentration was pronouncedly higher in the HFD group when compared with the control group (*p* < 0.01). However, the mixed lactobacilli administration markedly decreased the high levels of TG induced by high fat diet (*p* < 0.01).

### Effect of Mixed Lactobacilli on Oxidative Stress in the Liver

**FIGURE 4:**
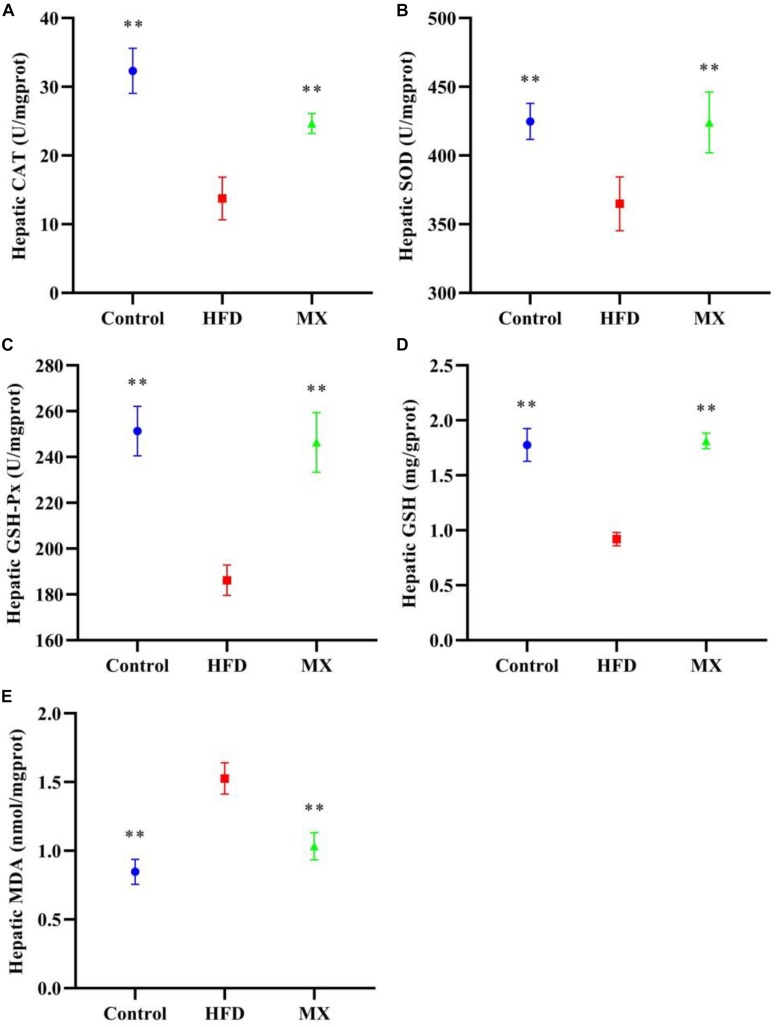
Effect of mixed lactobacilli on hepatic antioxidant enzymes, glutathione (GSH) and malondialdehyde (MDA). Control, control group; HFD, high fat diet group; and MX, mixed lactobacilli group. **(A)** Catalase (CAT); **(B)** Superoxide dismutase (SOD); **(C)** Glutathione peroxidase (GSH-Px); **(D)** GSH; and **(E)** MDA. All data are represented as mean ± SD. **p < 0.01: significant difference compared with mice in the HFD group.

The levels of antioxidant enzymes, GSH and MDA in the liver of the mice were measured. As depicted in Figures 4A–D, the levels of CAT, SOD, GSH-Px and GSH in the liver of the mice of the HFD group were significantly decreased as compared with that of the control group (*p* < 0.01). However, these reduced levels were significantly elevated by mixed lactobacilli supplementation (*p* < 0.01). Furthermore, mixed lactobacilli treatment significantly reduced liver MDA levels induced by high fat diet (Figure 4E, *p* < 0.01). These findings suggested that mixed lactobacilli administration could enhance the antioxidant capacity of the mice liver.

### Effect of Mixed Lactobacilli on Key Genes of Lipid Metabolism in Epididymal Fat Pads

**FIGURE 5:**
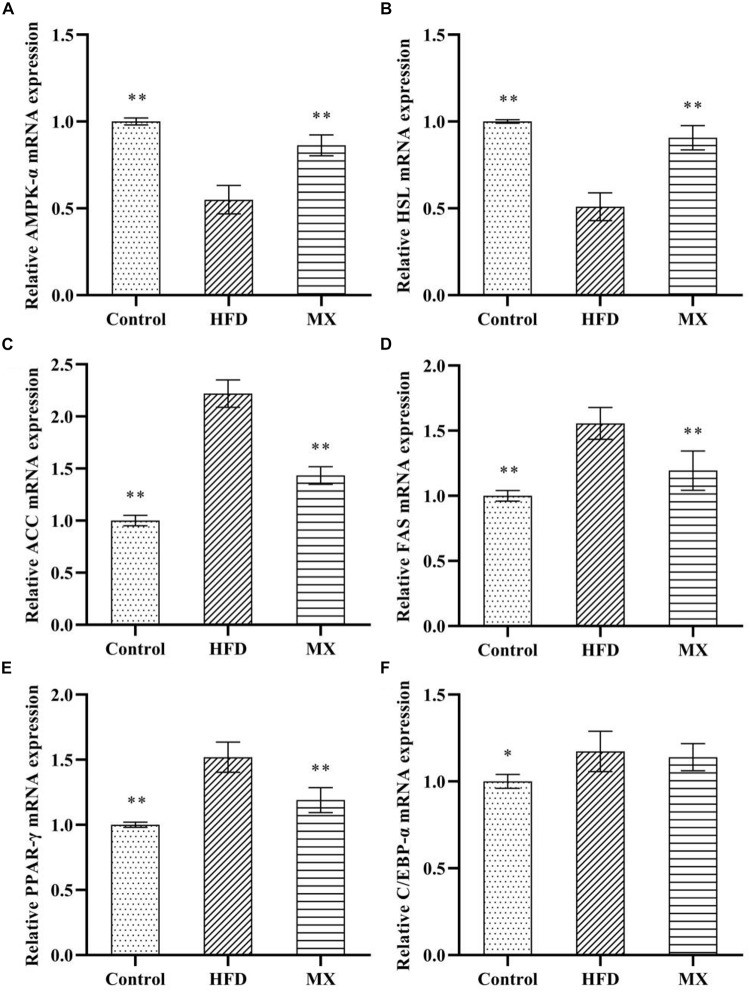
Effect of mixed lactobacilli on lipid metabolism regulating genes in epididymal fat pads. Control, control group; HFD, high fat diet group; and MX, mixed lactobacilli group. **(A)** Adenosine 5′-monophosphate-activated protein kinase-α (AMPK-α); **(B)** Hormone-sensitive lipase (HSL); **(C)** Acetyl CoA carboxylase (ACC); **(D)** Fatty acid synthetase (FAS); **(E)** Peroxisome proliferators-activated receptor-γ (PPAR-γ); and **(F)** CAATT/enhancer binding proteins α (C/EBPα). All data are represented as mean ± SD. ^∗^*p* < 0.05 and ^∗∗^*p* < 0.01: significant difference compared with mice in the HFD group.

The results and comparisons of mRNA expression of key genes for lipid metabolism in the epididymal fat pad are illustrated in Figure 5. As shown in Figures 5A,B, high fat diet feeding caused a significant down-regulation of mRNA levels of AMPK-α and HSL in the epididymal fat pad as compared to the control group (*p* < 0.01), which was normalized by the mixed lactobacilli supplementation. In addition, as shown in Figures 5C–F, the mRNA expression levels of ACC, FAS, PPAR-γ, and C/EBP-α in the epididymal fat pad of the mice in the HFD group were significantly higher than those in the control group (*p* < 0.01 or *p* < 0.05). However, after treatment with the mixed lactobacilli, the mRNA expression levels of ACC, FAS, and PPAR-γ were significantly decreased (*p* < 0.01), and the mRNA expression level of C/EBP-α was also decreased but not significant (*p* > 0.05).

### Effect of Mixed Lactobacilli on the Gut Microbiota Composition

**FIGURE 6:**
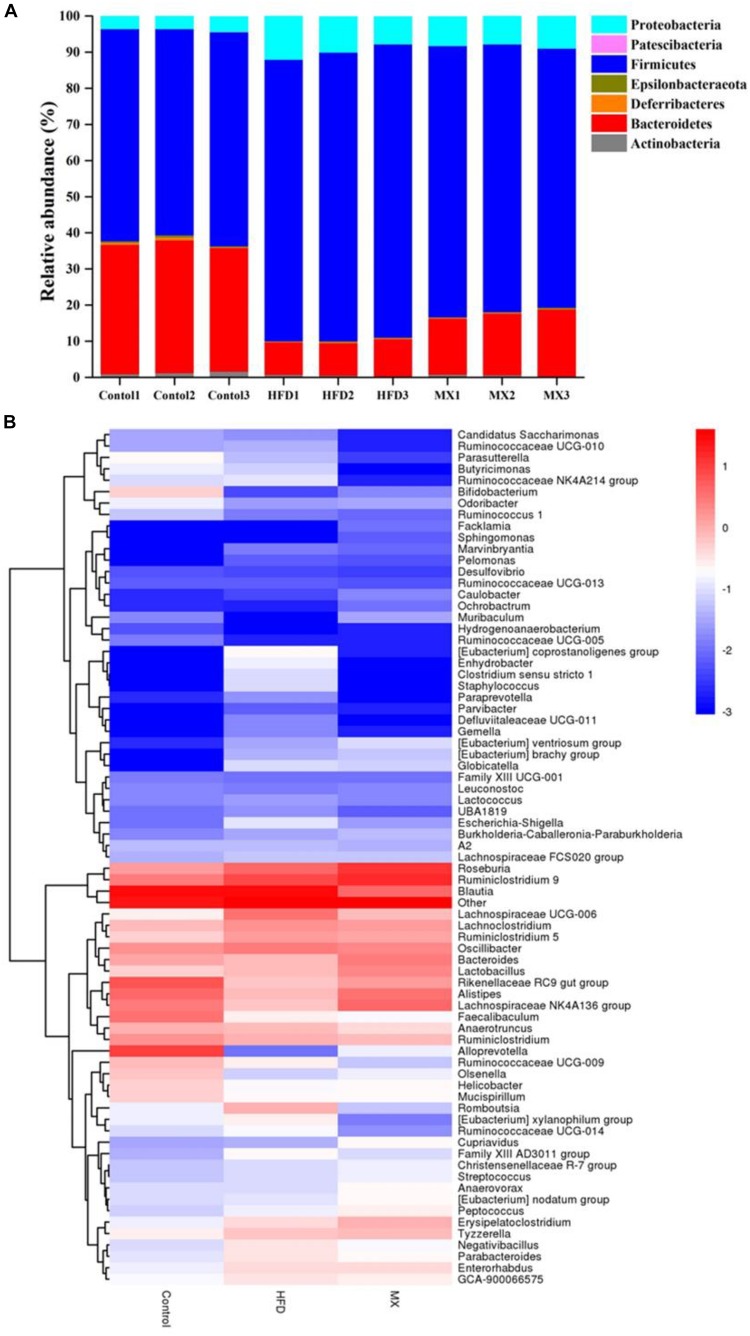
Changes of the gut microbial population at the phylum level following mixed lactobacilli administration. Control, control group; HFD, high fat diet group; and MX, mixed lactobacilli group. **(A)** Stacked bar plot of gut microbiota composition at the phylum level; **(B)** Heatmap of gut microbiota composition at the genus level. *n* = 3 per group.

To investigate whether mixed lactobacilli have an important role in the bacterial communities of high fat diet-fed mice, the cecal gut microbiota of the mice was analyzed by sequencing the 16S rDNA variable region V3-V4. At the phylum level, the dominant components in all groups were Firmicutes and Bacteroidetes, with a ratio of more than 85% (Figure 6A). Compared to the control group, the HFD group exhibited a higher relative abundance of Firmicutes and a lower relative abundance of Bacteroides, representing 79.67 and 9.43%, respectively. Consistently, a significant increase in the ratio of Firmicutes to Bacteroides in the HFD group was observed compared to the control group (*p* < 0.01, Table 1). However, mixed lactobacilli treatment attenuated the increase in Firmicutes, the decrease in Bacteroidetes and the increase in Firmicutes-to-Bacteroidetes ratio induced by high fat diet.

**TABLE 1:** Effect of mixed lactobacilli on the Firmicutes/Bacteroidetes ratio.

**Group**	**Firmicutes/Bacteroidetes ratio**
Control	1.64 ± 0.09**
HFD	8.47 ± 0.51
MX	4.39 ± 0.57**

Control, control group; HFD, high fat diet group; and MX, mixed lactobacilli group. All data are represented as mean ± SD. ** *p* < 0.01: significant difference compared with mice in the HFD group.

The distribution of gut microbiota at the genus level in different groups was shown by the genera abundance heatmap (Figure 6B). Compared with the control group, the relative abundances of *Bifidobacterium*, *Bacteroides*, *Alistipes*, *Lachnospiraceae NK4A136 group* and *Alloprevotella* were decreased in the HFD group but the relative abundances of *Parabacteroides*, *Eubacterium xylanophilum group*, *GCA-900066575*, *Lachnoclostridium*, *Lachnospiraceae UCG-006* and *Romboutsia* were increased, all of which were inhibited by mixed lactobacilli supplementation (Figure 6B). Collectively, these results implied that mixed lactobacilli consumption clearly modulated the taxonomic composition of the intestinal flora of mice fed with high fat diet.

**FIGURE 7:**
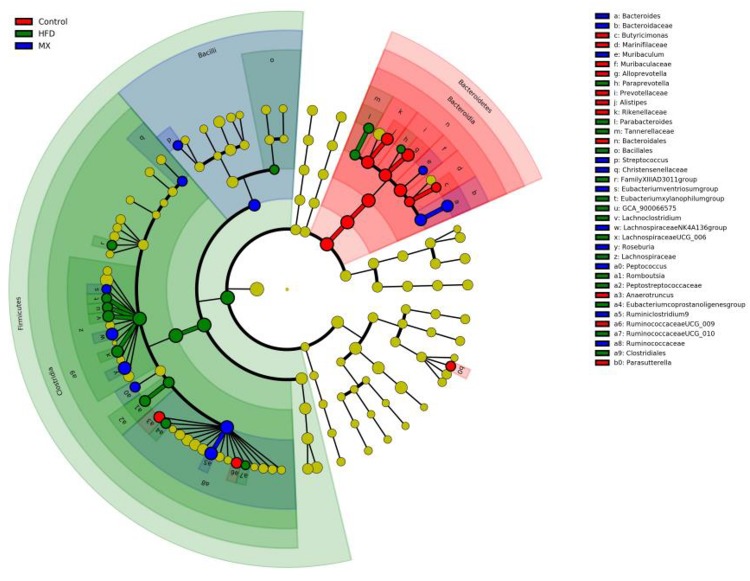
Linear Discriminant Analysis Effect Size (LEfSe) comparison of gut microbiota between the Control, HFD and MX groups. Control, control group; HFD, high fat diet group; and MX, mixed lactobacilli group. *n* = 3 per group.

**FIGURE 8:**
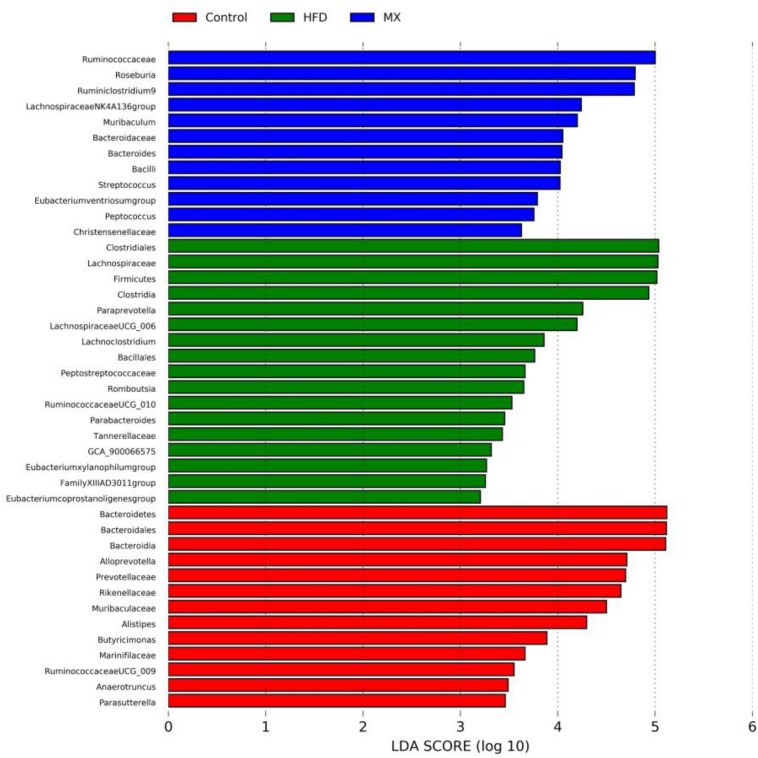
The Latent Dirichlet Allocation (LDA) scores indicate the effect size and ranking of each differentially abundant taxon between the Control, HFD and MX groups. Control, control group; HFD, high fat diet group; and MX, mixed lactobacilli group. *n* = 3 per group.

To identify predominant microbiota in each group, LEfSe analysis was performed. The resulting cladogram (Figure 7) disclosed that Bacteroidetes, *Alloprevotella* and *Alistipes* were more dominant in the control group than the other two groups. The HFD group was enriched with Firmicutes, *Lachnospiraceae UCG-006*, *Lachnoclostridium*, *Romboutsia*, *Parabacteroides*, *GCA-900066575* and *Eubacterium xylanophilum group*, while the MX group was enriched with *Lachnospiraceae NK4A136 group* and *Bacteroides*. The histogram of the Latent Dirichlet Allocation (LDA) scores (Figure 8) further revealed a clear difference between the control, HFD and MX groups in terms of the composition of biological clades, which was in agreement with the aforementioned results.

### Effect of Mixed Lactobacilli on SCFAs

To explore changes in SCFAs metabolism in the intestine, the levels of acetic acid, butyric acid, propionic acid and valeric acid in the cecal contents of different groups of mice were determined by GC-MS (Supplementary Figure S1). As shown in Table 2, the levels of acetic acid and butyric acid were strikingly decreased in the HFD group compared with the control group (*p* < 0.01), whereas supplement with mixed lactobacilli significantly increased the levels of the two SCFAs (*p* < 0.05 and *p* < 0.01). However, after 8 weeks of mixed lactobacilli treatment, the levels of propionic acid and valeric acid did not change significantly.

**TABLE 2:** The concentration of SCFAs in the mice intestine of different groups.

**Indices**	**Control**	**HFD**	**MX**
Acetic acid (mg/kg)	485.34 ± 33.18**	309.59 ± 10.20	380.56 ± 37.62*
Butyric acid (mg/kg)	449.87 ± 53.06**	267.99 ± 42.34	455.89 ± 15.02**
Propionic acid (mg/kg)	232.53 ± 32.61*	140.32 ± 22.79	133.36 ± 31.04
Valeric acid (mg/kg)	91.40 ± 14.42	72.90 ± 10.29	88.69 ± 6.79

Control, control group; HFD, high fat diet group; and MX, mixed lactobacilli group. All data are represented as mean ± SD. * *p* < 0.05 and ** *p* < 0.01: significant difference compared with mice in the HFD group.

### Correlation Between the Gut Microbiome and Obesity-Related Indicators

**FIGURE 9:**
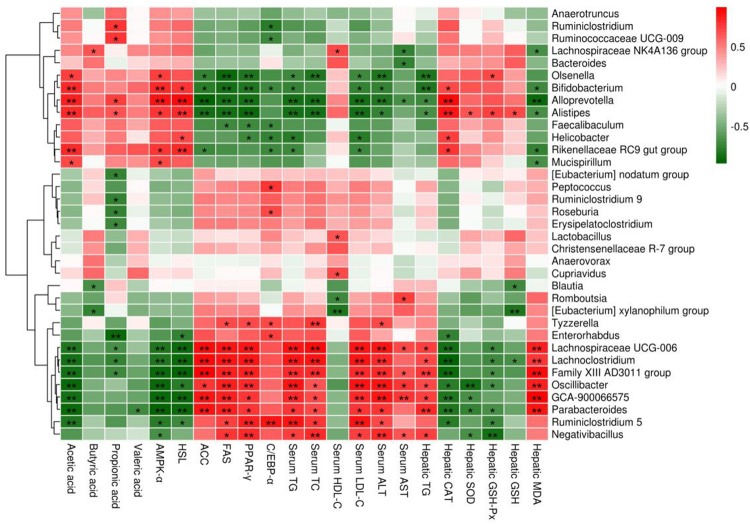
Heatmap of Spearman’s correlation between the relative abundance of gut microbiome at the genus level and obesity-related indicators. Short chain fatty acids: acetic acid, butyric acid, propionic acid and valeric acid; genes of epididymal adipose tissue: adenosine 5′-monophosphate-activated protein kinase-α (AMPK-α), hormone-sensitive lipase (HSL), acetyl CoA carboxylase (ACC), fatty acid synthetase (FAS), peroxisome proliferators-activated receptor-γ (PPAR-γ) and CAATT/enhancer binding proteins α (C/EBPα); hepatic parameters: triglyceride (TG), catalase (CAT), superoxide dismutase (SOD), glutathione peroxidase (GSH-Px), glutathione (GSH), malondialdehyde (MDA), alanine aminotransferase (ALT) and aspartate aminotransferase (AST); blood lipids: TG, total cholesterol (TC), high density lipoprotein cholesterol (HDL-C) and low density lipoprotein cholesterol (LDL-C). Significant correlations are indicated by *p < 0.05 and **p < 0.01.

The correlations between the relative abundances of the dominant gut microbial community at the genus level (the top 35 genera according to the relative abundance) and obesity-related indicators were determined by Spearman’s correlation analysis (Figure 9). Obesity-related indicators included short chain fatty acids (acetic acid, butyric acid, propionic acid and valeric acid), genes of epididymal adipose tissue (AMPK-α, HSL, ACC, FAS, PPAR-γ, and C/EBP-α), hepatic parameters (TG, CAT, SOD, GSH-Px, GSH, MDA, ALT, and AST) and blood lipids (TG, TC, HDL-C, and LDL-C). The relative abundances of *Bifidobacterium*, *Bacteroides*, *Alistipes*, *Lachnospiraceae NK4A136 group* and *Alloprevotella* were positively correlated with the levels of acetic acid and butyric acid, while the relative abundances of *Parabacteroides*, *Eubacterium xylanophilum group*, *GCA-900066575*, *Lachnoclostridium*, *Lachnospiraceae UCG-006* and *Romboutsia* were negatively correlated with the levels of acetic acid and butyric acid. Moreover, the relative abundances of *Bifidobacterium*, *Alistipes* and *Alloprevotella* showed significant positive correlations with the mRNA expression levels of AMPK-α and HSL (*p* < 0.01 or *p* < 0.05) and significant negative correlations with the mRNA expression levels of ACC, FAS and PPAR-γ (*p* < 0.01 or *p* < 0.05), but the relative abundances of *Parabacteroides*, *GCA-900066575*, *Lachnoclostridium* and *Lachnospiraceae UCG-006* showed the opposite trend (*p* < 0.01 or *p* < 0.05). In addition, *Bifidobacterium*, *Bacteroides*, *Alistipes*, *Lachnospiraceae NK4A136 group* and *Alloprevotella* were negatively correlated with TG and MDA in the liver and TG, TC, LDL-C, ALT and AST in the serum, whereas they were positively correlated with CAT, SOD, GSH-Px and GSH in the liver and HDL-C in the serum, but *Parabacteroides*, *Eubacterium xylanophilum group*, *GCA-900066575*, *Lachnoclostridium*, *Lachnospiraceae UCG-006* and *Romboutsia* were opposite.

## Discussion

Obesity, a metabolic disease, is becoming more common and is prone to other metabolic complications (such as cardiovascular disease and type 2 diabetes) (Sonnenburg and Backhed, 2016; Dahiya et al., 2017), pharmacotherapy causes adverse effects (Srivastava and Apovian, 2018; Rosa-Gonçalves and Majerowicz, 2019), so it is imperative to seek natural and non-toxic anti-obesity substances. Lactic acid bacteria have been used in fermented dairy products for more than 100 years (Aryana and Olson, 2017) and are generally regarded as safe (GRAS) (Özogul and Hamed, 2017). Moreover, to date, a large amount of evidence has shown that some lactic acid bacteria have an effective anti-obesity effect in animal research and clinical research (Dahiya et al., 2017). Previous researches in our laboratory have demonstrated that *L. plantarum* KLDS1.0344 and *L. plantarum* KLDS1.0386 have strong acid and bile salt resistance, high cell adhesion activities and lipid metabolism regulation properties (Tang et al., 2016; Jin et al., 2017; Lu et al., 2018; Yan et al., 2019; Yue et al., 2019). Therefore, further researches were carried out and it was found that mixed lactobacilli (*L. plantarum* KLDS1.0344 and *L. plantarum* KLDS1.0386) could prevent the formation of obesity in high fat diet-fed mice (Lu J. et al., 2019), which confirmed the probiotic properties of the mixed lactobacilli. However, the underlying mechanisms were unclear. Thus, in this study, the possible mechanisms by which the same lactobacilli strains could prevent obesity were mined.

The anti-obesity effect of mixed lactobacilli *in vivo* was studied using the D12492 high fat diet-induced obesity model, which is a widely used model for obesity research. Convincing evidence has demonstrated that obesity is often accompanied by dyslipidemia, such as elevated levels of TC, TG, and LDL-C, as well as decreased HDL-C levels, which are risk factors for cardiovascular disease (Hunter and Hegele, 2017; Kotsis et al., 2017). Thus, after 8 weeks of animal feeding, the levels of TC, TG, LDL-C and HDL-C in the serum of the three groups of mice were measured to evaluate their blood lipid metabolism. Our data indicated that compared with the control group, the serum levels of TC, TG and LDL-C were significantly increased, and the serum levels of HDL-C were significantly decreased in the HFD group, as expected. However, such changes in mice fed a high fat diet were reversed by the mixed lactobacilli treatment, implying an improvement in metabolic dysfunction. The findings were in agreement with previous research that a probiotic *L. plantarum* strain isolated from the homemade kumiss could effectively inhibit serum TC, TG, LDL-C and HDL-C changes from feeding with high fat diet (Wang et al., 2012).

Obesity is frequently characterized by the development of non-alcoholic fatty liver disease (NAFLD) (Michelotti et al., 2013; Canfora et al., 2019). The liver, an important site of lipid metabolism in the body, maintains the balance of lipid synthesis and decomposition under normal conditions, whereas a high-fat diet breaks this balance, causing excessive lipid accumulation (that is, hepatic steatosis) and oxidative stress in the liver, indicating the occurrence of liver injury (Rotman and Sanyal, 2017; Friedman et al., 2018). On this account, we aimed to determine the preventive effect of mixed lactobacilli intervention on NAFLD in high fat diet-fed mice. First, hepatic lipid accumulation was evaluated by measuring TG concentration. A significant elevated TG concentration in the liver was observed in the HFD group, which was consistent with the large number of lipid droplets in liver histopathological sections of the HFD group observed in our previous studies (Lu J. et al., 2019), implying that hepatic steatosis occurred. These results were in agreement with the earlier reports in which, obese mice suffered from non-alcoholic hepatic steatosis (Liou et al., 2019). However, it is noteworthy that mixed lactobacilli treatment substantially attenuated hepatic steatosis. Second, we assessed liver oxidative stress by analyzing antioxidant indices and lipid peroxidation biomarkers, including SOD, CAT, GSH-Px, GSH, and MDA. SOD, a critical antioxidant, can convert superoxide radical anions (O_2_^–^, incompletely reduced forms of oxygen) into hydrogen peroxide (H_2_O_2_), which in turn is catalyzed into water by CAT and GSH-Px (Turrens, 2003; Borrelli et al., 2018). GSH as a non-enzymatic antioxidant can directly scavenge reactive oxygen species (ROS) by binding with them (Anu et al., 2014). MDA is the end product of free radical-mediated lipid peroxidation and is currently considered a reliable biomarker related to oxidative stress (Wang et al., 2013). Our results demonstrated that oral administration of mixed lactobacilli significantly increased levels of SOD, CAT, GSH-Px, and GSH while significantly reducing MDA levels in high fat diet-fed mice, suggesting amelioration of liver oxidative stress. Finally, serum ALT and AST levels, often used to determine the extent of liver function damage (Zhao et al., 2017a), were measured. We found that serum levels of ALT and AST were significantly decreased in the MX group. Similarly, it has been previously stated that the treatment of the probiotic mixture (6 *Lactobacillus* and 3 *Bifidobacterium*) reduced serum ALT and AST levels in high fat diet-fed rats (Liang et al., 2018). Taken together, the mixed lactobacilli could inhibit liver lipid accumulation, enhance liver antioxidant capacity and improve liver function.

To further explore the potential mechanisms by which mixed lactobacilli inhibited obesity induced by high fat diet in mice, we examined the expression of lipid metabolism-related genes in epididymal adipose tissue. Adipose tissue, as one of the main sites for storing triglycerides, is an important organ regulating lipid metabolism (Schneeberger et al., 2015; Park et al., 2017). Extensive amounts of reports have shown that PPAR-γ and C/EBP-α are key transcription factors for adipocyte differentiation in adipose tissue (Prestwich and Macdougald, 2007). Park et al. (2014) demonstrated that *L. plantarum* LG42 supplementation strikingly decreased mRNA expression of PPAR-γ and C/EBP-α in high fat diet-fed mice. In this study, compared with HFD group, mixed lactobacilli treatment significantly down-regulated PPAR-γ mRNA levels, and also lowered the mRNA level of C/EBP-α but not significant. The AMPK pathway is a classical pathway that regulates lipid metabolism. AMPK is a known cellular energy sensor that shuts down anabolic pathways such as fatty acid synthesis (Hardie and Pan, 2002). ACC and FAS are key enzymes in fatty acid synthesis (Liou et al., 2019). Specifically, activation of AMPK-α stimulates ACC phosphorylation, which blocks the expression of FAS (Liou et al., 2019). HSL is the rate-limiting enzyme in the breakdown of triglycerides in adipose tissue (Liou et al., 2019). In the present study, the mRNA levels of AMPK-α and HSL were remarkably reduced in the HFD group compared with the control group, while the mRNA levels of ACC and FAS were significantly elevated, and these changes were completely eliminated by the treatment of mixed lactobacilli, similar to the study by Qiao et al. (Qiao et al., 2015).

Accumulating evidence suggests that the gut microbiota is a key environmental factor in the development of obesity (Walker and Julian, 2013). For instance, a previous study showed that germ-free, lean mice transplanted with intestinal microbiota from obese mice became obese, while those transplanted with intestinal flora from lean mice remained lean (Turnbaugh et al., 2006). Accordingly, gut microbiota is considered as a new target for the prevention and treatment of obesity. Subsequently, to investigate whether the mixed lactobacilli exerted its anti-obesity effects also through regulating the gut microbiota, we determined the intestinal bacteria composition of the three groups of mice. At the phylum level, we observed that the relative abundance of Firmicutes increased and the relative abundance of Bacteroidetes decreased in the HFD group compared with that in the control group, resulting in an significant increase in the ratio of Firmicutes/Bacteroidetes, which is consistent with many previous reports (Ilseung et al., 2012; Chang et al., 2015; Zhang et al., 2018). The researchers found that Firmicutes could produce more harvestable energy than Bacteroidetes, so in the case of an increase in the relative abundance of Firmicutes and a decrease in the relative abundance of Bacteroidetes, the absorption of calories increased and then promoted obesity (Turnbaugh et al., 2006; Komaroff, 2017). Noteworthily, the mixed lactobacilli supplementation in high fat diet-fed mice attenuated the increase in Firmicutes, the decrease in Bacteroidetes and the increase in the ratio of Firmicutes/Bacteroidetes. At the genus level, our results reflected that the relative abundances of *Bifidobacterium*, *Bacteroides*, *Alistipes*, *Lachnospiraceae NK4A136 group* and *Alloprevotella* decreased while that of *Parabacteroides*, *Eubacterium xylanophilum group*, *GCA-900066575*, *Lachnoclostridium*, *Lachnospiraceae UCG-006* and *Romboutsia* increased in the HFD group compared with the control group. In line with our results, earlier studies found that the abundances of *Bifidobacterium* (Zhu et al., 2018), *Bacteroides* (Zhang et al., 2015; Martinez et al., 2017), *Alistipes* (Zhu et al., 2018), *Lachnospiraceae NK4A136 group* (Hu et al., 2019), and *Alloprevotella* (Wang et al., 2019) were negatively correlated with obesity, and the abundances of *Parabacteroides* (Lieber et al., 2018; Zhao et al., 2018), *Eubacterium xylanophilum group* (Zhu J. et al., 2019), *GCA-900066575* (Zhu J. et al., 2019), *Lachnoclostridium* (Zhao et al., 2017b), *Lachnospiraceae UCG-006* (Kang et al., 2019), and *Romboutsia* (Zhao et al., 2018) were positively correlated with obesity. *Bifidobacterium*, a beneficial microbial species, producing lactic acid and acetic acid, can reduce intestinal pH and inhibit the growth of various detrimental bacteria to maintain intestinal health (Wang R. et al., 2018). *Bacteroides*, *Alistipes*, *Lachnospiraceae NK4A136 group* and *Alloprevotella* are also capable of producing SCFAs such as acetic acid and butyric acid (Borton et al., 2017; Gotoh et al., 2017; Jiang et al., 2018; Wang J. et al., 2018; Yin et al., 2018). It was reported that the abundance of *Bacteroides* was reduced in individuals with atherosclerotic cardiovascular disease (Jie et al., 2017) and post-inflammatory irritable bowel syndrome (Chen et al., 2017). *Alistipes* can effectively inhibit inflammation via preventing LPS-induced TNF-α release at higher concentrations (Canfora et al., 2015) and has been found at lower levels in the gut of patients with hepatocellular carcinoma (Ren et al., 2019), colitis (Jiang et al., 2018) and non-alcoholic fatty liver disease (Tang et al., 2018). Previous studies have indicated that *Lachnospiraceae NK4A136 group* may have an anti-inflammatory effect (Li et al., 2019) and its relative abundance was reduced in mice with immune dysfunction caused by cyclophosphamide (Zhu G. et al., 2019). *Alloprevotella* has been shown to be significantly reduced in mice with metabolic syndrome (Shang et al., 2017), a disease easily caused by obesity. According to previous studies, *Parabacteroides* was enriched in individuals with type 2 diabetes (Qin et al., 2012) and Behcet’s disease (Ye et al., 2018). *Eubacterium xylanophilum group*, *GCA-900066575*, *Lachnoclostridium*, *Lachnospiraceae UCG-006* and *Romboutsia* belong to Lachnospiraceae, which may suppress the growth of SCFA-producing bacteria (Duncan et al., 2002) and were found to be associated with metabolic disorders and colon cancer (Keishi and Kikuji, 2014; Meehan and Beiko, 2014). Importantly, the mixed lactobacilli treatment reversed the changes in several of the above genus.

Changes in the gut microbiota cause changes in the SCFAs levels, which are negatively correlated with obesity (Coelho et al., 2018). Lu Y. et al. (2016) have shown that supplementation of SCFAs in the diet dramatically inhibited the body weight gain in high fat diet-fed mice. SCFAs produced by bacteria may induce the release of gut-derived satiety hormones, such as peptide YY (PYY) and glucagon-like peptide-1 (GLP-1), which suppress food intake and increase satiety (Canfora et al., 2015). Furthermore, studies have indicated that SCFAs can reach the liver through the portal vein, thereby activating the nuclear erythroid 2-related factor 2 (Nrf-2) pathway to alleviate oxidative stress (Li et al., 2018) and activating the AMPK pathway to inhibit lipid accumulation (Canfora et al., 2015). In addition, SCFAs have been reported to regulate lipid metabolism in adipose tissue by modulating related signaling pathways (Gijs et al., 2015). Therefore, we determined the levels of acetic acid, butyric acid, propionic acid and valeric acid in each group by GC-MS. Intriguingly, compared to the HFD group, mixed lactobacilli supplementation significantly increased the concentrations of acetic acid and butyric acid, which was in agreement with changes in the intestinal microbiota. Based on the above results, we speculated that the mixed lactobacilli treatment might attenuate liver oxidative stress, reduce liver lipid accumulation and improve lipid metabolism of adipose tissue by regulating intestinal microbiota and metabolites. In line with our finding, Yin et al. (2018) concluded that one of the potential mechanisms of melatonin inhibiting obesity might related to the increased levels of acetic acid and butyric acid.

To further confirm the role of gut microbiota in anti-obesity, we also analyzed the correlation between the relative abundance of mixed lactobacilli-manipulated gut microbiota and obesity-related indicators through Spearman’s correlation analysis. In general, *Bifidobacterium*, *Bacteroides*, *Alistipes*, *Lachnospiraceae NK4A136 group* and *Alloprevotella* were positively correlated with acetic acid and butyric acid in intestine, AMPK-α and HSL in epididymal adipose tissue, CAT, SOD, GSH-Px and GSH in liver, and HDL-C in serum, while they were negatively correlated with ACC, FAS and PPAR-γ in epididymal adipose tissue, TG and MDA in liver, and TG, TC, LDL-C, ALT and AST in serum, but *Parabacteroides*, *Eubacterium xylanophilum group*, *GCA-900066575*, *Lachnoclostridium*, *Lachnospiraceae UCG-006* and *Romboutsia* displayed the opposite trend. Therefore, the results also indicated that mixed lactobacilli administration could effectively modulate the gut microbiota induced by high fat diet, and then improve obesity-related indicators.

## Conclusion

The mixed lactobacilli intervention could alleviate the changes induced by high fat diet including disordered blood lipids, liver oxidative stress and liver injury. Further, the mixed lactobacilli intervention modulated the gut microbiota of high fat diet-fed mice, resulting in increased SCFAs (acetic acid and butyric acid), which regulated lipid metabolism in adipose tissue and reduced liver lipid accumulation, thereby preventing obesity. Hence, our results offer significant insight into the oral administration of mixed lactobacilli to suppress obesity in high fat diet-fed mice.

## Data Availability Statement

All datasets generated for this study are included in the article/Supplementary Material.

## Ethics Statement

The animal study was reviewed and approved by the Institutional Animal Care and Use Committee of the Northeast Agricultural University.

## Author Contributions

HL did the experiments and wrote the manuscript. FL and JL did the experiments. JS, JG, and FY processed the data. BL and GH corrected the manuscript.

## Conflict of Interest

The authors declare that the research was conducted in the absence of any commercial or financial relationships that could be construed as a potential conflict of interest.
