# Supplementary material for: Probiotic Mixture of Lactobacillus plantarum Strains Improves Lipid Metabolism and Gut Microbiota Structure in High Fat Diet-Fed Mice
Source: Front Microbiol. 2020 Mar 26;11:512. doi: 10.3389/fmicb.2020.00512 (PMC7113563; doi:10.3389/fmicb.2020.00512)
Supplement: Supplementary file 1 [file Data_Sheet_1.docx]

**Table S1** Feed formula of mice

| Ingredient | D12450B control feed (g/kg) | D12492 high fat feed (g/kg) |
| --- | --- | --- |
| Casein, 80mesh | 200 | 200 |
| L-Cystine | 3 | 3 |
| Corn starch | 315 | 0 |
| Maltodextrin 10 | 35 | 125 |
| Sucrose | 350 | 68.8 |
| Cellulose BW20 | 50 | 50 |
| Soybean oil | 25 | 25 |
| Lard | 20 | 245 |
| Mineral mix S10026 | 10 | 10 |
| Dicalcium phosphate | 13 | 13 |
| Calcium carbonate | 5.5 | 5.5 |
| Potassium citrate, 1 H_2_O | 16.5 | 16.5 |
| Vitamin mix V10001 | 10 | 10 |
| Choline bitartrate | 2 | 2 |
| FD&C yellow dye # 5 | 0.05 |  |
| FD&C blue dye # 1 |  | 0.05 |

**Table S2** Forward and reverse primer sequences for quantitative real-time PCR.

| Gene | Forward sequence | Reverse sequence |
| --- | --- | --- |
| AMPKα | 5’-AGTTCGAGTGTTCGGAGGAGGAG-3’ | 5’-GAGGTGGTAGGCGACGGCTAG-3’ |
| HSL  PPAR-γ  C/EBPα | 5’-GCACGGCGGCTGTCTAATGTC-3’  5’-CCATCGAGGACATCCAAGACAACC-3’  5’-AAGTCGGTGGACAAGAACAGCAAC-3’ | 5’-CGTTGGCTGGTGTCTCTGTGTC-3’  5’-GGAGCACCTTGGCGAACAGC-3’  5’-CGGTCATTGTCACTGGTCAACTCC-3’ |
| FAS | 5’-TGCACCTCGTGTGAACATGG-3’ | 5’-ATGGTCAGCAACCATAGGCG-3’ |
| ACC | 5’-TTTCAGTTCATGCTGCCCACA-3’ | 5’-AGGTTGGAGGCAAAGGACAT-3’ |
| β-actin | 5’-GGGTCAGAAGGACTCCTATG-3’ | 5’-GTAACAATGCCATGTTCAAT-3’ |


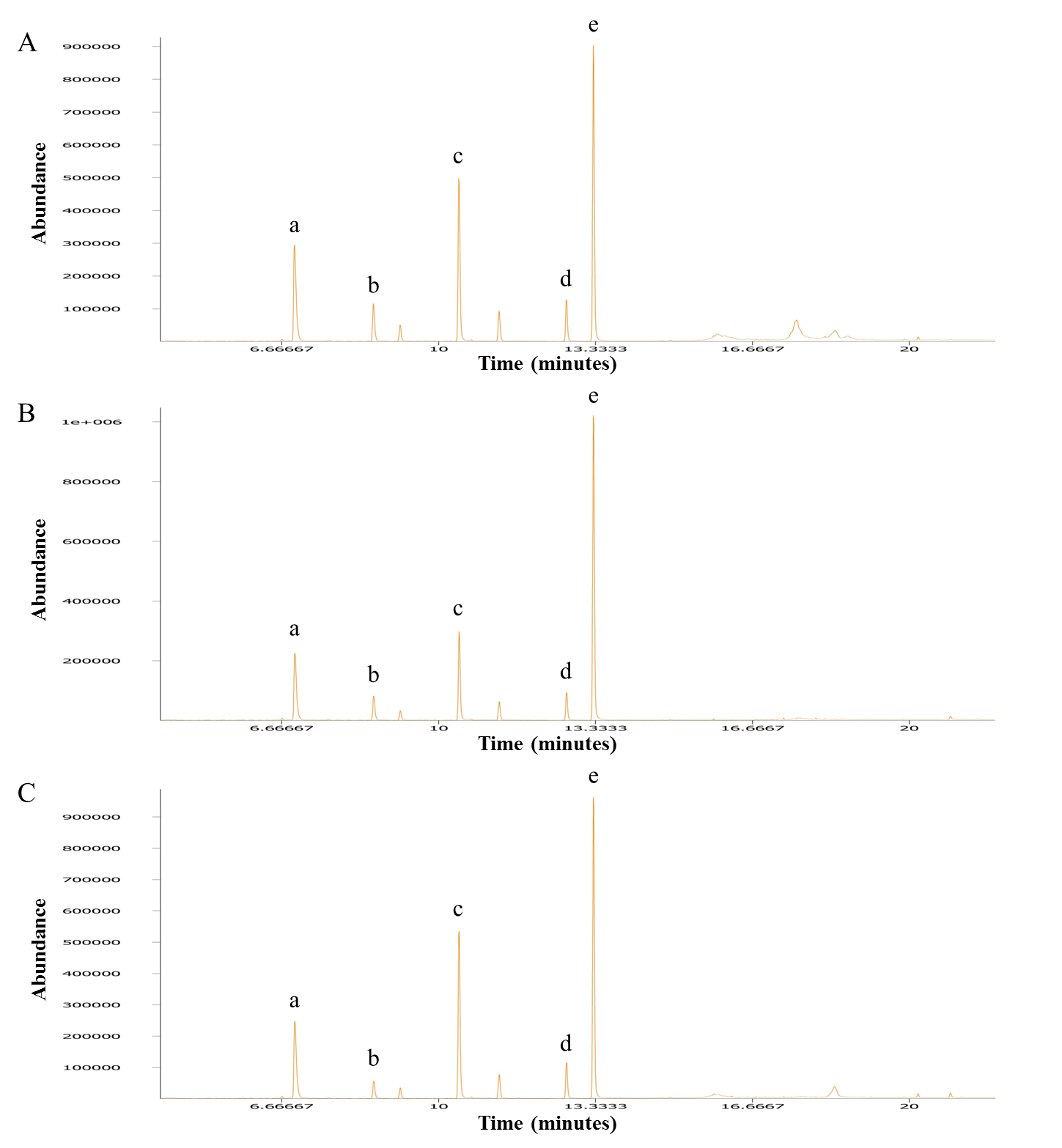


**Figure S1** Representative GC-MS chromatograms. (A) Control group (Control); (B) High fat diet group (HFD); and (C) Mixed lactobacilli group (MX). (a) Acetic acid; (b) Propionic acid; (c) Butyric acid; (d) Valeric acid; and (e) 2-Methylvaleric acid (Internal standard).
